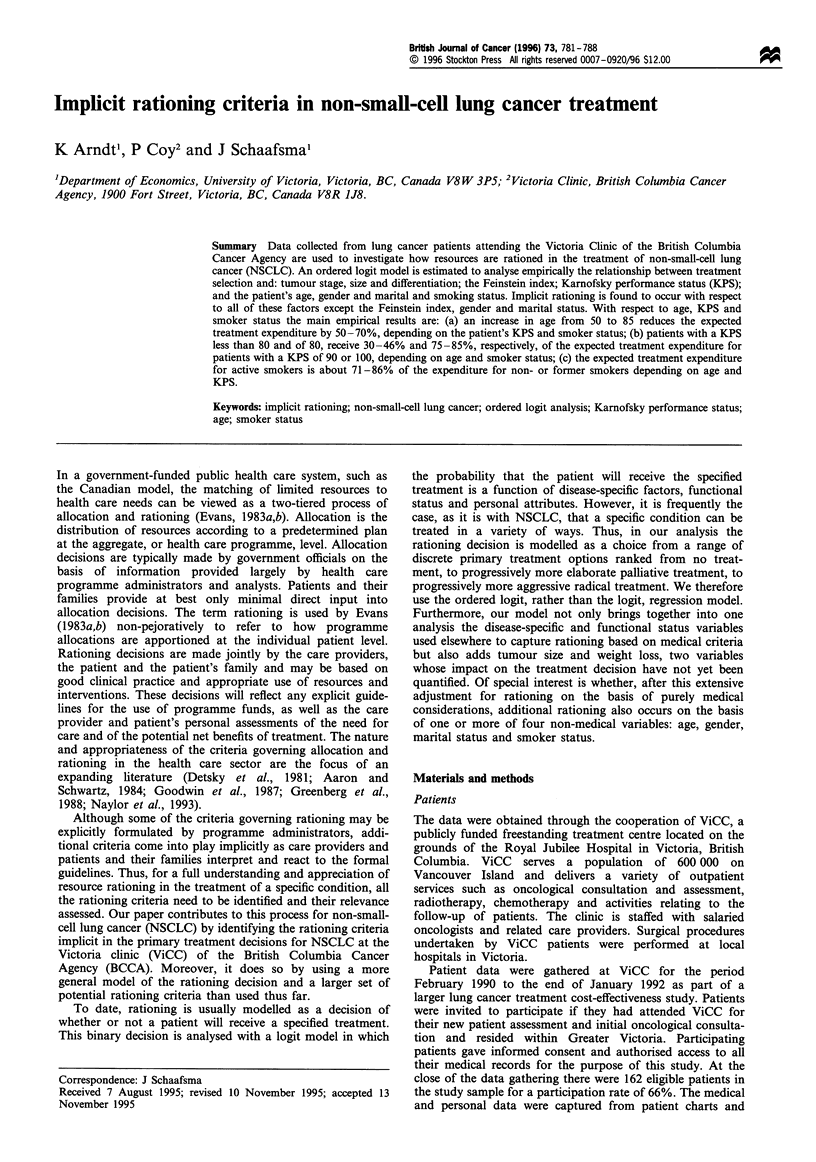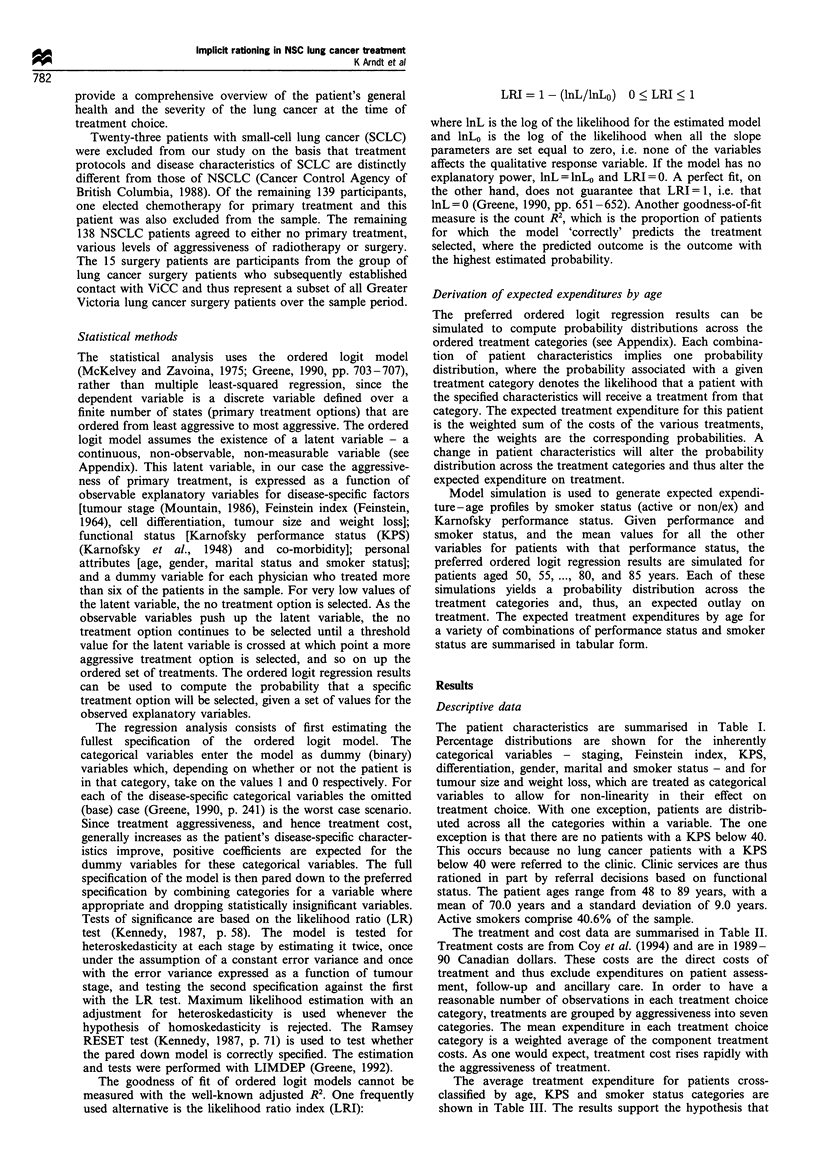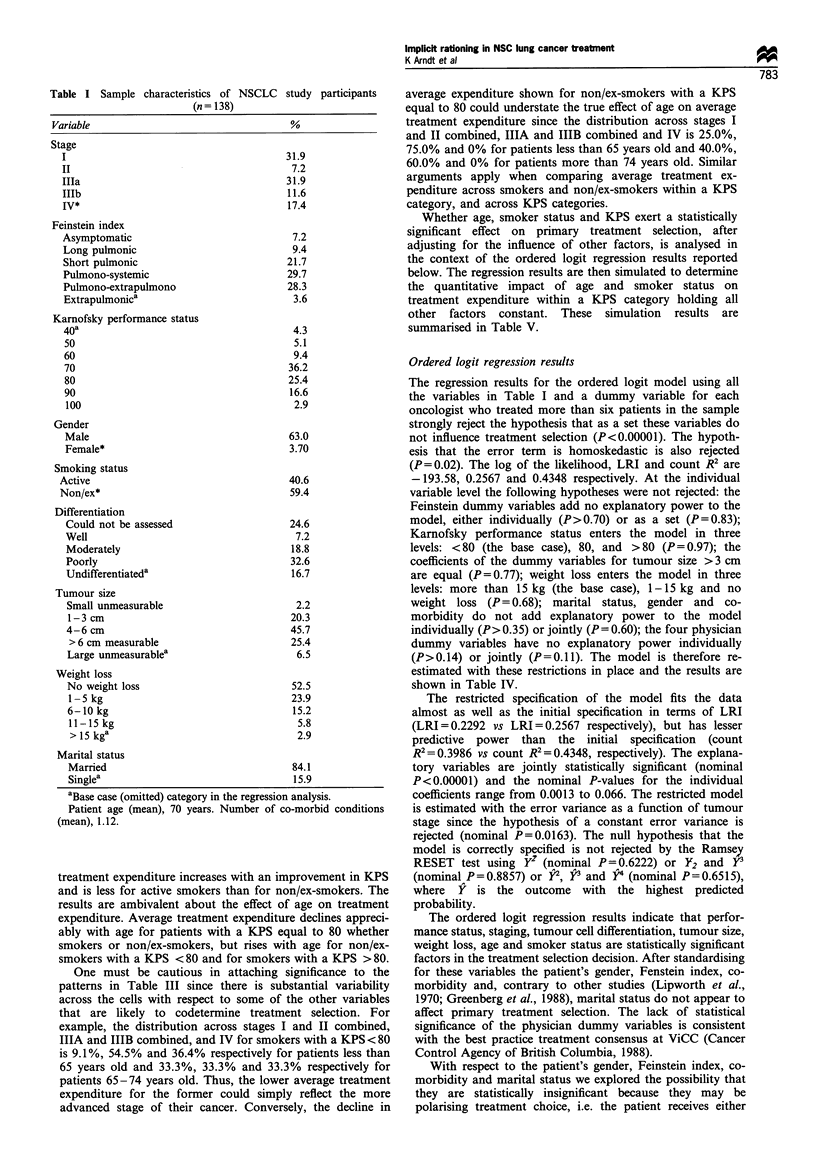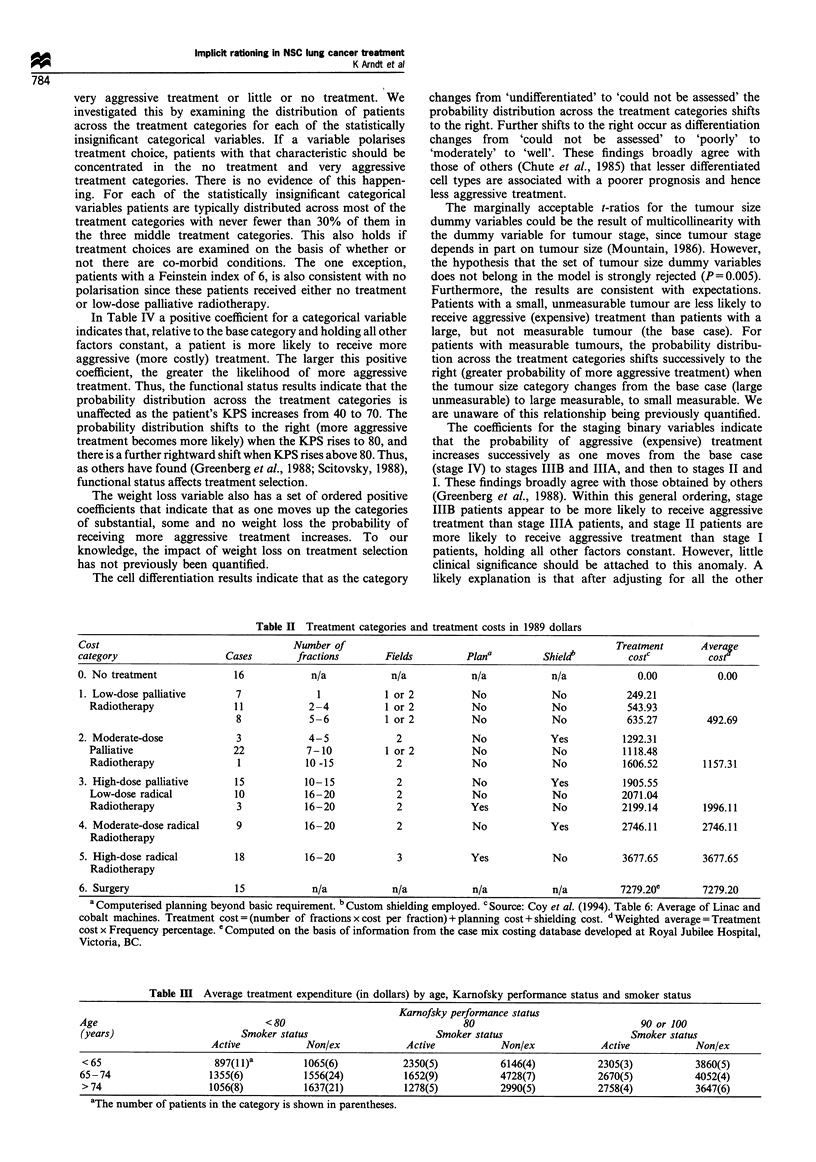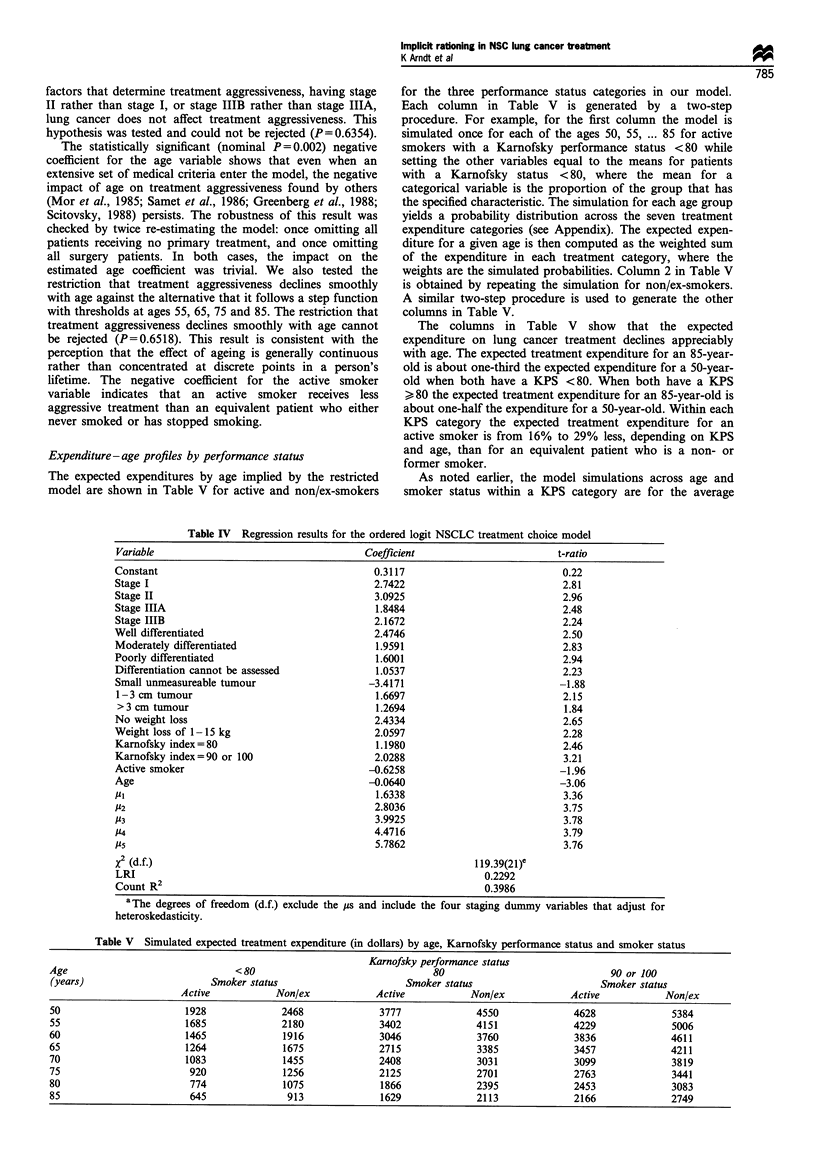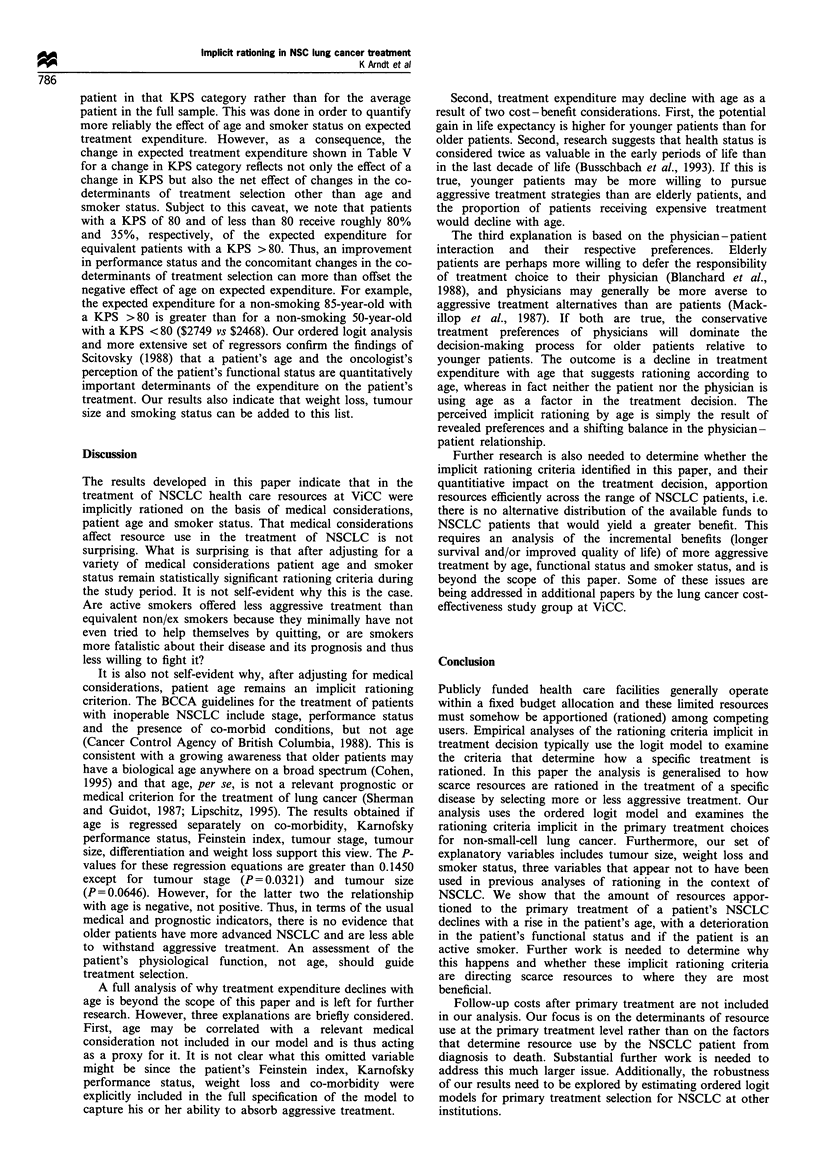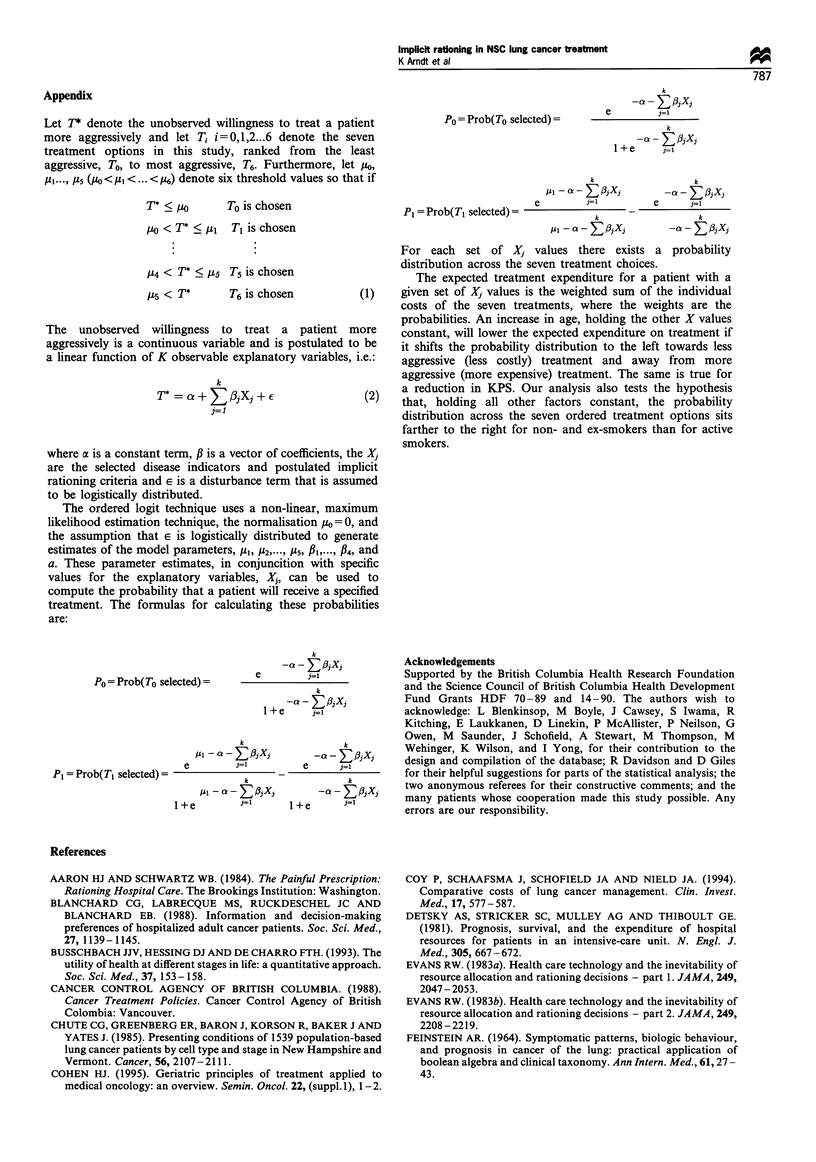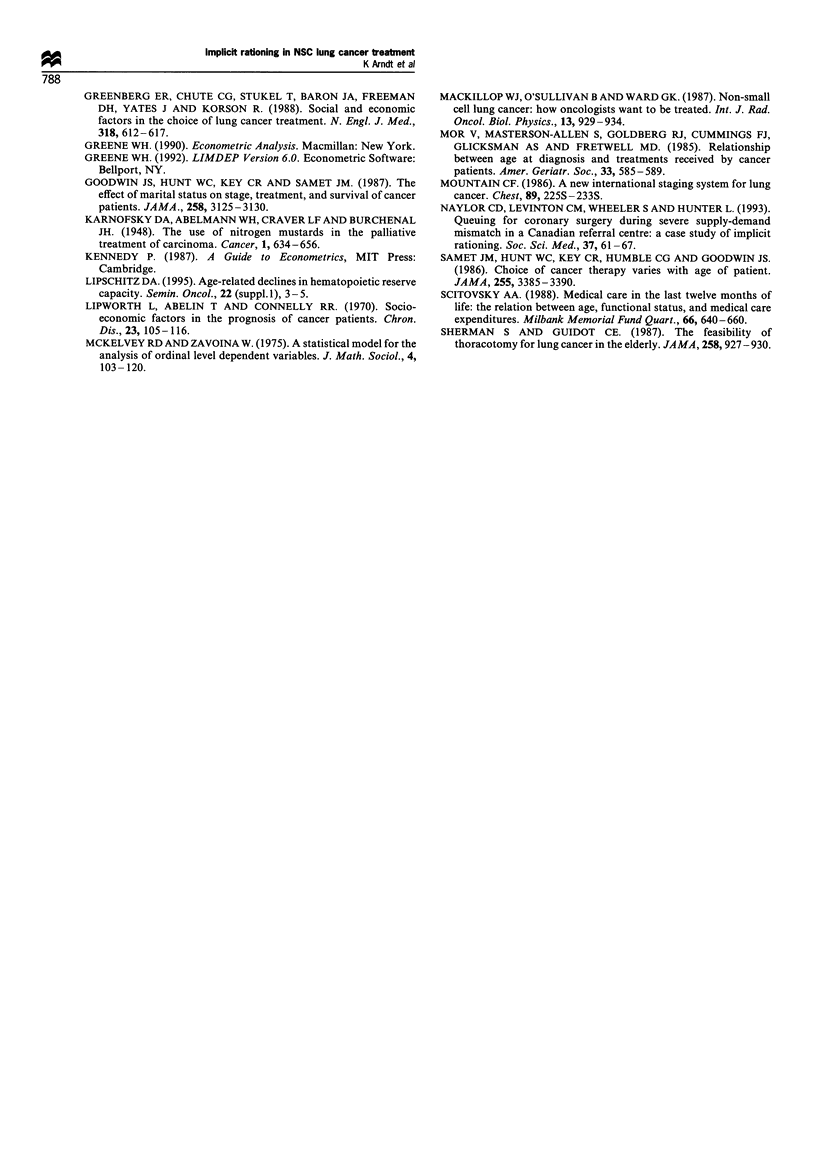# Implicit rationing criteria in non-small-cell lung cancer treatment.

**DOI:** 10.1038/bjc.1996.136

**Published:** 1996-03

**Authors:** K. Arndt, P. Coy, J. Schaafsma

**Affiliations:** Department of Economics, University of Victoria, Canada.

## Abstract

Data collected from lung cancer patients attending the Victoria Clinic of the British Columbia Cancer Agency are used to investigate how resources are rationed in the treatment of non-small-cell lung cancer (NSCLC). An ordered logit model is estimated to analyse empirically the relationship between treatment selection and: tumour stage, size and differentiation; the Feinstein index; Karnofsky performance status (KPS); and the patient's age, gender and marital and smoking status. Implicit rationing is found to occur with respect to all of these factors except the Feinstein index, gender and marital status. With respect to age, KPS and smoker status the main empirical results are: (a) an increase in age from 50 to 85 reduces the expected treatment expenditure by 50-70%, depending on the patient's KPS and smoker status; (b) patients with a KPS less than 80 and of 80, receive 30-46% and 75-85%, respectively, of the expected treatment expenditure for patients with a KPS of 90 or 100, depending on age and smoker status; (c) the expected treatment expenditure for active smokers is about 71-86% of the expenditure for non- or former smokers depending on age and KPS.


British Journal of Cancer (1996) 73, 781-788
© 1996 Stockton Press All rights reserved 0007-0920/96 $12.00

### Implicit rationing criteria in non-small-cell lung cancer treatment

K Arndt[1], P Coy[2] and J Schaafsma[1]

[1]*Department of Economics, University of Victoria, Victoria, BC, Canada V8W 3P5;* [2]*Victoria Clinic, British Columbia Cancer Agency, 1900 Fort Street, Victoria, BC, Canada V8R 1J8.*

**Summary** Data collected from lung cancer patients attending the Victoria Clinic of the British Columbia Cancer Agency are used to investigate how resources are rationed in the treatment of non-small-cell lung cancer (NSCLC). An ordered logit model is estimated to analyse empirically the relationship between treatment selection and: tumour stage, size and differentiation; the Feinstein index; Karnofsky performance status (KPS); and the patient's age, gender and marital and smoking status. Implicit rationing is found to occur with respect to all of these factors except the Feinstein index, gender and marital status. With respect to age, KPS and smoker status the main empirical results are: (a) an increase in age from 50 to 85 reduces the expected treatment expenditure by 50–70%, depending on the patient's KPS and smoker status; (b) patients with a KPS less than 80 and of 80, receive 30–46% and 75–85%, respectively, of the expected treatment expenditure for patients with a KPS of 90 or 100, depending on age and smoker status; (c) the expected treatment expenditure for active smokers is about 71–86% of the expenditure for non- or former smokers depending on age and KPS.

**Keywords:** implicit rationing; non-small-cell lung cancer; ordered logit analysis; Karnofsky performance status; age; smoker status

In a government-funded public health care system, such as the Canadian model, the matching of limited resources to health care needs can be viewed as a two-tiered process of allocation and rationing (Evans, 1983*a,b*). Allocation is the distribution of resources according to a predetermined plan at the aggregate, or health care programme, level. Allocation decisions are typically made by government officials on the basis of information provided largely by health care programme administrators and analysts. Patients and their families provide at best only minimal direct input into allocation decisions. The term rationing is used by Evans (1983*a,b*) non-pejoratively to refer to how programme allocations are apportioned at the individual patient level. Rationing decisions are made jointly by the care providers, the patient and the patient's family and may be based on good clinical practice and appropriate use of resources and interventions. These decisions will reflect any explicit guidelines for the use of programme funds, as well as the care provider and patient's personal assessments of the need for care and of the potential net benefits of treatment. The nature and appropriateness of the criteria governing allocation and rationing in the health care sector are the focus of an expanding literature (Detsky *et al.*, 1981; Aaron and Schwartz, 1984; Goodwin *et al.*, 1987; Greenberg *et al.*, 1988; Naylor *et al.*, 1993).

Although some of the criteria governing rationing may be explicitly formulated by programme administrators, additional criteria come into play implicitly as care providers and patients and their families interpret and react to the formal guidelines. Thus, for a full understanding and appreciation of resource rationing in the treatment of a specific condition, all the rationing criteria need to be identified and their relevance assessed. Our paper contributes to this process for non-small-cell lung cancer (NSCLC) by identifying the rationing criteria implicit in the primary treatment decisions for NSCLC at the Victoria clinic (ViCC) of the British Columbia Cancer Agency (BCCA). Moreover, it does so by using a more general model of the rationing decision and a larger set of potential rationing criteria than used thus far.

To date, rationing is usually modelled as a decision of whether or not a patient will receive a specified treatment. This binary decision is analysed with a logit model in which the probability that the patient will receive the specified treatment is a function of disease-specific factors, functional status and personal attributes. However, it is frequently the case, as it is with NSCLC, that a specific condition can be treated in a variety of ways. Thus, in our analysis the rationing decision is modelled as a choice from a range of discrete primary treatment options ranked from no treatment, to progressively more elaborate palliative treatment, to progressively more aggressive radical treatment. We therefore use the ordered logit, rather than the logit, regression model. Furthermore, our model not only brings together into one analysis the disease-specific and functional status variables used elsewhere to capture rationing based on medical criteria but also adds tumour size and weight loss, two variables whose impact on the treatment decision have not yet been quantified. Of special interest is whether, after this extensive adjustment for rationing on the basis of purely medical considerations, additional rationing also occurs on the basis of one or more of four non-medical variables: age, gender, marital status and smoker status.

### Materials and methods

### *Patients*

The data were obtained through the cooperation of ViCC, a publicly funded freestanding treatment centre located on the grounds of the Royal Jubilee Hospital in Victoria, British Columbia. ViCC serves a population of 600 000 on Vancouver Island and delivers a variety of outpatient services such as oncological consultation and assessment, radiotherapy, chemotherapy and activities relating to the follow-up of patients. The clinic is staffed with salaried oncologists and related care providers. Surgical procedures undertaken by ViCC patients were performed at local hospitals in Victoria.

Patient data were gathered at ViCC for the period February 1990 to the end of January 1992 as part of a larger lung cancer treatment cost-effectiveness study. Patients were invited to participate if they had attended ViCC for their new patient assessment and initial oncological consultation and resided within Greater Victoria. Participating patients gave informed consent and authorised access to all their medical records for the purpose of this study. At the close of the data gathering there were 162 eligible patients in the study sample for a participation rate of 66%. The medical and personal data were captured from patient charts and

Correspondence: J Schaafsma
Received 7 August 1995; revised 10 November 1995; accepted 13 November 1995

provide a comprehensive overview of the patient's general health and the severity of the lung cancer at the time of treatment choice.

Twenty-three patients with small-cell lung cancer (SCLC) were excluded from our study on the basis that treatment protocols and disease characteristics of SCLC are distinctly different from those of NSCLC (Cancer Control Agency of British Columbia, 1988). Of the remaining 139 participants, one elected chemotherapy for primary treatment and this patient was also excluded from the sample. The remaining 138 NSCLC patients agreed to either no primary treatment, various levels of aggressiveness of radiotherapy or surgery. The 15 surgery patients are participants from the group of lung cancer surgery patients who subsequently established contact with ViCC and thus represent a subset of all Greater Victoria lung cancer surgery patients over the sample period.

*Statistical methods*

The statistical analysis uses the ordered logit model (McKelvey and Zavoina, 1975; Greene, 1990, pp. 703–707), rather than multiple least-squared regression, since the dependent variable is a discrete variable defined over a finite number of states (primary treatment options) that are ordered from least aggressive to most aggressive. The ordered logit model assumes the existence of a latent variable – a continuous, non-observable, non-measurable variable (see Appendix). This latent variable, in our case the aggressiveness of primary treatment, is expressed as a function of observable explanatory variables for disease-specific factors [tumour stage (Mountain, 1986), Feinstein index (Feinstein, 1964), cell differentiation, tumour size and weight loss]; functional status [Karnofsky performance status (KPS) (Karnofsky *et al.*, 1948) and co-morbidity]; personal attributes [age, gender, marital status and smoker status]; and a dummy variable for each physician who treated more than six of the patients in the sample. For very low values of the latent variable, the no treatment option is selected. As the observable variables push up the latent variable, the no treatment option continues to be selected until a threshold value for the latent variable is crossed at which point a more aggressive treatment option is selected, and so on up the ordered set of treatments. The ordered logit regression results can be used to compute the probability that a specific treatment option will be selected, given a set of values for the observed explanatory variables.

The regression analysis consists of first estimating the fullest specification of the ordered logit model. The categorical variables enter the model as dummy (binary) variables which, depending on whether or not the patient is in that category, take on the values 1 and 0 respectively. For each of the disease-specific categorical variables the omitted (base) case (Greene, 1990, p. 241) is the worst case scenario. Since treatment aggressiveness, and hence treatment cost, generally increases as the patient's disease-specific characteristics improve, positive coefficients are expected for the dummy variables for these categorical variables. The full specification of the model is then pared down to the preferred specification by combining categories for a variable where appropriate and dropping statistically insignificant variables. Tests of significance are based on the likelihood ratio (LR) test (Kennedy, 1987, p. 58). The model is tested for heteroskedasticity at each stage by estimating it twice, once under the assumption of a constant error variance and once with the error variance expressed as a function of tumour stage, and testing the second specification against the first with the LR test. Maximum likelihood estimation with an adjustment for heteroskedasticity is used whenever the hypothesis of homoskedasticity is rejected. The Ramsey RESET test (Kennedy, 1987, p. 71) is used to test whether the pared down model is correctly specified. The estimation and tests were performed with LIMDEP (Greene, 1992).

The goodness of fit of ordered logit models cannot be measured with the well-known adjusted $R^2$. One frequently used alternative is the likelihood ratio index (LRI):

$$\mathrm{LRI} = 1 - (\ln L/\ln L_0) \quad 0 \leq \mathrm{LRI} \leq 1$$

where lnL is the log of the likelihood for the estimated model and $\ln L_0$ is the log of the likelihood when all the slope parameters are set equal to zero, i.e. none of the variables affects the qualitative response variable. If the model has no explanatory power, $\ln L = \ln L_0$ and LRI = 0. A perfect fit, on the other hand, does not guarantee that LRI = 1, i.e. that lnL = 0 (Greene, 1990, pp. 651–652). Another goodness-of-fit measure is the count $R^2$, which is the proportion of patients for which the model 'correctly' predicts the treatment selected, where the predicted outcome is the outcome with the highest estimated probability.

*Derivation of expected expenditures by age*

The preferred ordered logit regression results can be simulated to compute probability distributions across the ordered treatment categories (see Appendix). Each combination of patient characteristics implies one probability distribution, where the probability associated with a given treatment category denotes the likelihood that a patient with the specified characteristics will receive a treatment from that category. The expected treatment expenditure for this patient is the weighted sum of the costs of the various treatments, where the weights are the corresponding probabilities. A change in patient characteristics will alter the probability distribution across the treatment categories and thus alter the expected expenditure on treatment.

Model simulation is used to generate expected expenditure–age profiles by smoker status (active or non/ex) and Karnofsky performance status. Given performance and smoker status, and the mean values for all the other variables for patients with that performance status, the preferred ordered logit regression results are simulated for patients aged 50, 55, ..., 80, and 85 years. Each of these simulations yields a probability distribution across the treatment categories and, thus, an expected outlay on treatment. The expected treatment expenditures by age for a variety of combinations of performance status and smoker status are summarised in tabular form.

### Results

*Descriptive data*

The patient characteristics are summarised in Table I. Percentage distributions are shown for the inherently categorical variables – staging, Feinstein index, KPS, differentiation, gender, marital and smoker status – and for tumour size and weight loss, which are treated as categorical variables to allow for non-linearity in their effect on treatment choice. With one exception, patients are distributed across all the categories within a variable. The one exception is that there are no patients with a KPS below 40. This occurs because no lung cancer patients with a KPS below 40 were referred to the clinic. Clinic services are thus rationed in part by referral decisions based on functional status. The patient ages range from 48 to 89 years, with a mean of 70.0 years and a standard deviation of 9.0 years. Active smokers comprise 40.6% of the sample.

The treatment and cost data are summarised in Table II. Treatment costs are from Coy *et al.* (1994) and are in 1989–90 Canadian dollars. These costs are the direct costs of treatment and thus exclude expenditures on patient assessment, follow-up and ancillary care. In order to have a reasonable number of observations in each treatment choice category, treatments are grouped by aggressiveness into seven categories. The mean expenditure in each treatment choice category is a weighted average of the component treatment costs. As one would expect, treatment cost rises rapidly with the aggressiveness of treatment.

The average treatment expenditure for patients cross-classified by age, KPS and smoker status categories are shown in Table III. The results support the hypothesis that

Table I Sample characteristics of NSCLC study participants ($n=138$)

| *Variable* | % |
|---|---|
| Stage | |
| I | 31.9 |
| II | 7.2 |
| IIIa | 31.9 |
| IIIb | 11.6 |
| IV* | 17.4 |
| Feinstein index | |
| Asymptomatic | 7.2 |
| Long pulmonic | 9.4 |
| Short pulmonic | 21.7 |
| Pulmono-systemic | 29.7 |
| Pulmono-extrapulmono | 28.3 |
| Extrapulmonic[a] | 3.6 |
| Karnofsky performance status | |
| 40[a] | 4.3 |
| 50 | 5.1 |
| 60 | 9.4 |
| 70 | 36.2 |
| 80 | 25.4 |
| 90 | 16.6 |
| 100 | 2.9 |
| Gender | |
| Male | 63.0 |
| Female* | 3.70 |
| Smoking status | |
| Active | 40.6 |
| Non/ex* | 59.4 |
| Differentiation | |
| Could not be assessed | 24.6 |
| Well | 7.2 |
| Moderately | 18.8 |
| Poorly | 32.6 |
| Undifferentiated[a] | 16.7 |
| Tumour size | |
| Small unmeasurable | 2.2 |
| 1-3 cm | 20.3 |
| 4-6 cm | 45.7 |
| >6 cm measurable | 25.4 |
| Large unmeasurable[a] | 6.5 |
| Weight loss | |
| No weight loss | 52.5 |
| 1-5 kg | 23.9 |
| 6-10 kg | 15.2 |
| 11-15 kg | 5.8 |
| >15 kg[a] | 2.9 |
| Marital status | |
| Married | 84.1 |
| Single[a] | 15.9 |

[a]Base case (omitted) category in the regression analysis.
Patient age (mean), 70 years. Number of co-morbid conditions (mean), 1.12.

treatment expenditure increases with an improvement in KPS and is less for active smokers than for non/ex-smokers. The results are ambivalent about the effect of age on treatment expenditure. Average treatment expenditure declines appreciably with age for patients with a KPS equal to 80 whether smokers or non/ex-smokers, but rises with age for non/ex-smokers with a KPS <80 and for smokers with a KPS >80.

One must be cautious in attaching significance to the patterns in Table III since there is substantial variability across the cells with respect to some of the other variables that are likely to codetermine treatment selection. For example, the distribution across stages I and II combined, IIIA and IIIB combined, and IV for smokers with a KPS<80 is 9.1%, 54.5% and 36.4% respectively for patients less than 65 years old and 33.3%, 33.3% and 33.3% respectively for patients 65-74 years old. Thus, the lower average treatment expenditure for the former could simply reflect the more advanced stage of their cancer. Conversely, the decline in average expenditure shown for non/ex-smokers with a KPS equal to 80 could understate the true effect of age on average treatment expenditure since the distribution across stages I and II combined, IIIA and IIIB combined and IV is 25.0%, 75.0% and 0% for patients less than 65 years old and 40.0%, 60.0% and 0% for patients more than 74 years old. Similar arguments apply when comparing average treatment expenditure across smokers and non/ex-smokers within a KPS category, and across KPS categories.

Whether age, smoker status and KPS exert a statistically significant effect on primary treatment selection, after adjusting for the influence of other factors, is analysed in the context of the ordered logit regression results reported below. The regression results are then simulated to determine the quantitative impact of age and smoker status on treatment expenditure within a KPS category holding all other factors constant. These simulation results are summarised in Table V.

### *Ordered logit regression results*

The regression results for the ordered logit model using all the variables in Table I and a dummy variable for each oncologist who treated more than six patients in the sample strongly reject the hypothesis that as a set these variables do not influence treatment selection ($P<0.00001$). The hypothesis that the error term is homoskedastic is also rejected ($P=0.02$). The log of the likelihood, LRI and count $R^2$ are $-193.58$, 0.2567 and 0.4348 respectively. At the individual variable level the following hypotheses were not rejected: the Feinstein dummy variables add no explanatory power to the model, either individually ($P>0.70$) or as a set ($P=0.83$); Karnofsky performance status enters the model in three levels: <80 (the base case), 80, and >80 ($P=0.97$); the coefficients of the dummy variables for tumour size >3 cm are equal ($P=0.77$); weight loss enters the model in three levels: more than 15 kg (the base case), 1-15 kg and no weight loss ($P=0.68$); marital status, gender and co-morbidity do not add explanatory power to the model individually ($P>0.35$) or jointly ($P=0.60$); the four physician dummy variables have no explanatory power individually ($P>0.14$) or jointly ($P=0.11$). The model is therefore re-estimated with these restrictions in place and the results are shown in Table IV.

The restricted specification of the model fits the data almost as well as the initial specification in terms of LRI (LRI=0.2292 *vs* LRI=0.2567 respectively), but has lesser predictive power than the initial specification (count $R^2=0.3986$ *vs* count $R^2=0.4348$, respectively). The explanatory variables are jointly statistically significant (nominal $P<0.00001$) and the nominal $P$-values for the individual coefficients range from 0.0013 to 0.066. The restricted model is estimated with the error variance as a function of tumour stage since the hypothesis of a constant error variance is rejected (nominal $P=0.0163$). The null hypothesis that the model is correctly specified is not rejected by the Ramsey RESET test using $\hat{Y}^2$ (nominal $P=0.6222$) or $\hat{Y}_2$ and $\hat{Y}^3$ (nominal $P=0.8857$) or $\hat{Y}^2$, $\hat{Y}^3$ and $\hat{Y}^4$ (nominal $P=0.6515$), where $\hat{Y}$ is the outcome with the highest predicted probability.

The ordered logit regression results indicate that performance status, staging, tumour cell differentiation, tumour size, weight loss, age and smoker status are statistically significant factors in the treatment selection decision. After standardising for these variables the patient's gender, Fenstein index, co-morbidity and, contrary to other studies (Lipworth *et al.*, 1970; Greenberg *et al.*, 1988), marital status do not appear to affect primary treatment selection. The lack of statistical significance of the physician dummy variables is consistent with the best practice treatment consensus at ViCC (Cancer Control Agency of British Columbia, 1988).

With respect to the patient's gender, Feinstein index, co-morbidity and marital status we explored the possibility that they are statistically insignificant because they may be polarising treatment choice, i.e. the patient receives either very aggressive treatment or little or no treatment. We investigated this by examining the distribution of patients across the treatment categories for each of the statistically insignificant categorical variables. If a variable polarises treatment choice, patients with that characteristic should be concentrated in the no treatment and very aggressive treatment categories. There is no evidence of this happening. For each of the statistically insignificant categorical variables patients are typically distributed across most of the treatment categories with never fewer than 30% of them in the three middle treatment categories. This also holds if treatment choices are examined on the basis of whether or not there are co-morbid conditions. The one exception, patients with a Feinstein index of 6, is also consistent with no polarisation since these patients received either no treatment or low-dose palliative radiotherapy.

In Table IV a positive coefficient for a categorical variable indicates that, relative to the base category and holding all other factors constant, a patient is more likely to receive more aggressive (more costly) treatment. The larger this positive coefficient, the greater the likelihood of more aggressive treatment. Thus, the functional status results indicate that the probability distribution across the treatment categories is unaffected as the patient's KPS increases from 40 to 70. The probability distribution shifts to the right (more aggressive treatment becomes more likely) when the KPS rises to 80, and there is a further rightward shift when KPS rises above 80. Thus, as others have found (Greenberg *et al.*, 1988; Scitovsky, 1988), functional status affects treatment selection.

The weight loss variable also has a set of ordered positive coefficients that indicate that as one moves up the categories of substantial, some and no weight loss the probability of receiving more aggressive treatment increases. To our knowledge, the impact of weight loss on treatment selection has not previously been quantified.

The cell differentiation results indicate that as the category changes from 'undifferentiated' to 'could not be assessed' the probability distribution across the treatment categories shifts to the right. Further shifts to the right occur as differentiation changes from 'could not be assessed' to 'poorly' to 'moderately' to 'well'. These findings broadly agree with those of others (Chute *et al.*, 1985) that lesser differentiated cell types are associated with a poorer prognosis and hence less aggressive treatment.

The marginally acceptable $t$-ratios for the tumour size dummy variables could be the result of multicollinearity with the dummy variable for tumour stage, since tumour stage depends in part on tumour size (Mountain, 1986). However, the hypothesis that the set of tumour size dummy variables does not belong in the model is strongly rejected ($P = 0.005$). Furthermore, the results are consistent with expectations. Patients with a small, unmeasurable tumour are less likely to receive aggressive (expensive) treatment than patients with a large, but not measurable tumour (the base case). For patients with measurable tumours, the probability distribution across the treatment categories shifts successively to the right (greater probability of more aggressive treatment) when the tumour size category changes from the base case (large unmeasurable) to large measurable, to small measurable. We are unaware of this relationship being previously quantified.

The coefficients for the staging binary variables indicate that the probability of aggressive (expensive) treatment increases successively as one moves from the base case (stage IV) to stages IIIB and IIIA, and then to stages II and I. These findings broadly agree with those obtained by others (Greenberg *et al.*, 1988). Within this general ordering, stage IIIB patients appear to be more likely to receive aggressive treatment than stage IIIA patients, and stage II patients are more likely to receive aggressive treatment than stage I patients, holding all other factors constant. However, little clinical significance should be attached to this anomaly. A likely explanation is that after adjusting for all the other

**Table II** Treatment categories and treatment costs in 1989 dollars

| *Cost category* | *Cases* | *Number of fractions* | *Fields* | *Plan*[a] | *Shield*[b] | *Treatment cost*[c] | *Average cost*[d] |
|---|---|---|---|---|---|---|---|
| 0. No treatment | 16 | n/a | n/a | n/a | n/a | 0.00 | 0.00 |
| 1. Low-dose palliative | 7 | 1 | 1 or 2 | No | No | 249.21 | |
| Radiotherapy | 11 | 2–4 | 1 or 2 | No | No | 543.93 | |
| | 8 | 5–6 | 1 or 2 | No | No | 635.27 | 492.69 |
| 2. Moderate-dose | 3 | 4–5 | 2 | No | Yes | 1292.31 | |
| Palliative | 22 | 7–10 | 1 or 2 | No | No | 1118.48 | |
| Radiotherapy | 1 | 10 -15 | 2 | No | No | 1606.52 | 1157.31 |
| 3. High-dose palliative | 15 | 10–15 | 2 | No | Yes | 1905.55 | |
| Low-dose radical | 10 | 16–20 | 2 | No | No | 2071.04 | |
| Radiotherapy | 3 | 16–20 | 2 | Yes | No | 2199.14 | 1996.11 |
| 4. Moderate-dose radical Radiotherapy | 9 | 16–20 | 2 | No | Yes | 2746.11 | 2746.11 |
| 5. High-dose radical Radiotherapy | 18 | 16–20 | 3 | Yes | No | 3677.65 | 3677.65 |
| 6. Surgery | 15 | n/a | n/a | n/a | n/a | 7279.20[e] | 7279.20 |

[a] Computerised planning beyond basic requirement. [b] Custom shielding employed. [c] Source: Coy *et al.* (1994). Table 6: Average of Linac and cobalt machines. Treatment cost = (number of fractions × cost per fraction) + planning cost + shielding cost. [d] Weighted average = Treatment cost × Frequency percentage. [e] Computed on the basis of information from the case mix costing database developed at Royal Jubilee Hospital, Victoria, BC.

**Table III** Average treatment expenditure (in dollars) by age, Karnofsky performance status and smoker status

| *Age (years)* | *Karnofsky performance status* <br> *<80* <br> *Smoker status* <br> *Active* | *Non/ex* | *80* <br> *Smoker status* <br> *Active* | *Non/ex* | *90 or 100* <br> *Smoker status* <br> *Active* | *Non/ex* |
|---|---|---|---|---|---|---|
| <65 | 897(11)[a] | 1065(6) | 2350(5) | 6146(4) | 2305(3) | 3860(5) |
| 65–74 | 1355(6) | 1556(24) | 1652(9) | 4728(7) | 2670(5) | 4052(4) |
| >74 | 1056(8) | 1637(21) | 1278(5) | 2990(5) | 2758(4) | 3647(6) |

[a] The number of patients in the category is shown in parentheses.

factors that determine treatment aggressiveness, having stage II rather than stage I, or stage IIIB rather than stage IIIA, lung cancer does not affect treatment aggressiveness. This hypothesis was tested and could not be rejected ($P=0.6354$).

The statistically significant (nominal $P=0.002$) negative coefficient for the age variable shows that even when an extensive set of medical criteria enter the model, the negative impact of age on treatment aggressiveness found by others (Mor *et al.*, 1985; Samet *et al.*, 1986; Greenberg *et al.*, 1988; Scitovsky, 1988) persists. The robustness of this result was checked by twice re-estimating the model: once omitting all patients receiving no primary treatment, and once omitting all surgery patients. In both cases, the impact on the estimated age coefficient was trivial. We also tested the restriction that treatment aggressiveness declines smoothly with age against the alternative that it follows a step function with thresholds at ages 55, 65, 75 and 85. The restriction that treatment aggressiveness declines smoothly with age cannot be rejected ($P=0.6518$). This result is consistent with the perception that the effect of ageing is generally continuous rather than concentrated at discrete points in a person's lifetime. The negative coefficient for the active smoker variable indicates that an active smoker receives less aggressive treatment than an equivalent patient who either never smoked or has stopped smoking.

*Expenditure–age profiles by performance status*

The expected expenditures by age implied by the restricted model are shown in Table V for active and non/ex-smokers for the three performance status categories in our model. Each column in Table V is generated by a two-step procedure. For example, for the first column the model is simulated once for each of the ages 50, 55, ... 85 for active smokers with a Karnofsky performance status <80 while setting the other variables equal to the means for patients with a Karnofsky status <80, where the mean for a categorical variable is the proportion of the group that has the specified characteristic. The simulation for each age group yields a probability distribution across the seven treatment expenditure categories (see Appendix). The expected expenditure for a given age is then computed as the weighted sum of the expenditure in each treatment category, where the weights are the simulated probabilities. Column 2 in Table V is obtained by repeating the simulation for non/ex-smokers. A similar two-step procedure is used to generate the other columns in Table V.

The columns in Table V show that the expected expenditure on lung cancer treatment declines appreciably with age. The expected treatment expenditure for an 85-year-old is about one-third the expected expenditure for a 50-year-old when both have a KPS <80. When both have a KPS ⩾80 the expected treatment expenditure for an 85-year-old is about one-half the expenditure for a 50-year-old. Within each KPS category the expected treatment expenditure for an active smoker is from 16% to 29% less, depending on KPS and age, than for an equivalent patient who is a non- or former smoker.

As noted earlier, the model simulations across age and smoker status within a KPS category are for the average

**Table IV** Regression results for the ordered logit NSCLC treatment choice model

| *Variable* | *Coefficient* | t-*ratio* |
|---|---|---|
| Constant | 0.3117 | 0.22 |
| Stage I | 2.7422 | 2.81 |
| Stage II | 3.0925 | 2.96 |
| Stage IIIA | 1.8484 | 2.48 |
| Stage IIIB | 2.1672 | 2.24 |
| Well differentiated | 2.4746 | 2.50 |
| Moderately differentiated | 1.9591 | 2.83 |
| Poorly differentiated | 1.6001 | 2.94 |
| Differentiation cannot be assessed | 1.0537 | 2.23 |
| Small unmeasureable tumour | −3.4171 | −1.88 |
| 1–3 cm tumour | 1.6697 | 2.15 |
| >3 cm tumour | 1.2694 | 1.84 |
| No weight loss | 2.4334 | 2.65 |
| Weight loss of 1–15 kg | 2.0597 | 2.28 |
| Karnofsky index = 80 | 1.1980 | 2.46 |
| Karnofsky index = 90 or 100 | 2.0288 | 3.21 |
| Active smoker | −0.6258 | −1.96 |
| Age | −0.0640 | −3.06 |
| $\mu_1$ | 1.6338 | 3.36 |
| $\mu_2$ | 2.8036 | 3.75 |
| $\mu_3$ | 3.9925 | 3.78 |
| $\mu_4$ | 4.4716 | 3.79 |
| $\mu_5$ | 5.7862 | 3.76 |
| $\chi^2$ (d.f.) | 119.39(21)[a] | |
| LRI | 0.2292 | |
| Count $R^2$ | 0.3986 | |

[a] The degrees of freedom (d.f.) exclude the $\mu$s and include the four staging dummy variables that adjust for heteroskedasticity.

**Table V** Simulated expected treatment expenditure (in dollars) by age, Karnofsky performance status and smoker status

| *Age (years)* | *Karnofsky performance status* <80 *Smoker status* | | 80 *Smoker status* | | 90 or 100 *Smoker status* | |
|---|---|---|---|---|---|---|
| | *Active* | *Non/ex* | *Active* | *Non/ex* | *Active* | *Non/ex* |
| 50 | 1928 | 2468 | 3777 | 4550 | 4628 | 5384 |
| 55 | 1685 | 2180 | 3402 | 4151 | 4229 | 5006 |
| 60 | 1465 | 1916 | 3046 | 3760 | 3836 | 4611 |
| 65 | 1264 | 1675 | 2715 | 3385 | 3457 | 4211 |
| 70 | 1083 | 1455 | 2408 | 3031 | 3099 | 3819 |
| 75 | 920 | 1256 | 2125 | 2701 | 2763 | 3441 |
| 80 | 774 | 1075 | 1866 | 2395 | 2453 | 3083 |
| 85 | 645 | 913 | 1629 | 2113 | 2166 | 2749 |

patient in that KPS category rather than for the average patient in the full sample. This was done in order to quantify more reliably the effect of age and smoker status on expected treatment expenditure. However, as a consequence, the change in expected treatment expenditure shown in Table V for a change in KPS category reflects not only the effect of a change in KPS but also the net effect of changes in the co-determinants of treatment selection other than age and smoker status. Subject to this caveat, we note that patients with a KPS of 80 and of less than 80 receive roughly 80% and 35%, respectively, of the expected expenditure for equivalent patients with a KPS >80. Thus, an improvement in performance status and the concomitant changes in the co-determinants of treatment selection can more than offset the negative effect of age on expected expenditure. For example, the expected expenditure for a non-smoking 85-year-old with a KPS >80 is greater than for a non-smoking 50-year-old with a KPS <80 ($2749 *vs* $2468). Our ordered logit analysis and more extensive set of regressors confirm the findings of Scitovsky (1988) that a patient's age and the oncologist's perception of the patient's functional status are quantitatively important determinants of the expenditure on the patient's treatment. Our results also indicate that weight loss, tumour size and smoking status can be added to this list.

### Discussion

The results developed in this paper indicate that in the treatment of NSCLC health care resources at ViCC were implicitly rationed on the basis of medical considerations, patient age and smoker status. That medical considerations affect resource use in the treatment of NSCLC is not surprising. What is surprising is that after adjusting for a variety of medical considerations patient age and smoker status remain statistically significant rationing criteria during the study period. It is not self-evident why this is the case. Are active smokers offered less aggressive treatment than equivalent non/ex smokers because they minimally have not even tried to help themselves by quitting, or are smokers more fatalistic about their disease and its prognosis and thus less willing to fight it?

It is also not self-evident why, after adjusting for medical considerations, patient age remains an implicit rationing criterion. The BCCA guidelines for the treatment of patients with inoperable NSCLC include stage, performance status and the presence of co-morbid conditions, but not age (Cancer Control Agency of British Columbia, 1988). This is consistent with a growing awareness that older patients may have a biological age anywhere on a broad spectrum (Cohen, 1995) and that age, *per se*, is not a relevant prognostic or medical criterion for the treatment of lung cancer (Sherman and Guidot, 1987; Lipschitz, 1995). The results obtained if age is regressed separately on co-morbidity, Karnofsky performance status, Feinstein index, tumour stage, tumour size, differentiation and weight loss support this view. The *P*-values for these regression equations are greater than 0.1450 except for tumour stage ($P=0.0321$) and tumour size ($P=0.0646$). However, for the latter two the relationship with age is negative, not positive. Thus, in terms of the usual medical and prognostic indicators, there is no evidence that older patients have more advanced NSCLC and are less able to withstand aggressive treatment. An assessment of the patient's physiological function, not age, should guide treatment selection.

A full analysis of why treatment expenditure declines with age is beyond the scope of this paper and is left for further research. However, three explanations are briefly considered. First, age may be correlated with a relevant medical consideration not included in our model and is thus acting as a proxy for it. It is not clear what this omitted variable might be since the patient's Feinstein index, Karnofsky performance status, weight loss and co-morbidity were explicitly included in the full specification of the model to capture his or her ability to absorb aggressive treatment.

Second, treatment expenditure may decline with age as a result of two cost–benefit considerations. First, the potential gain in life expectancy is higher for younger patients than for older patients. Second, research suggests that health status is considered twice as valuable in the early periods of life than in the last decade of life (Busschbach *et al.*, 1993). If this is true, younger patients may be more willing to pursue aggressive treatment strategies than are elderly patients, and the proportion of patients receiving expensive treatment would decline with age.

The third explanation is based on the physician–patient interaction and their respective preferences. Elderly patients are perhaps more willing to defer the responsibility of treatment choice to their physician (Blanchard *et al.*, 1988), and physicians may generally be more averse to aggressive treatment alternatives than are patients (Mackillop *et al.*, 1987). If both are true, the conservative treatment preferences of physicians will dominate the decision-making process for older patients relative to younger patients. The outcome is a decline in treatment expenditure with age that suggests rationing according to age, whereas in fact neither the patient nor the physician is using age as a factor in the treatment decision. The perceived implicit rationing by age is simply the result of revealed preferences and a shifting balance in the physician–patient relationship.

Further research is also needed to determine whether the implicit rationing criteria identified in this paper, and their quantitiative impact on the treatment decision, apportion resources efficiently across the range of NSCLC patients, i.e. there is no alternative distribution of the available funds to NSCLC patients that would yield a greater benefit. This requires an analysis of the incremental benefits (longer survival and/or improved quality of life) of more aggressive treatment by age, functional status and smoker status, and is beyond the scope of this paper. Some of these issues are being addressed in additional papers by the lung cancer cost-effectiveness study group at ViCC.

### Conclusion

Publicly funded health care facilities generally operate within a fixed budget allocation and these limited resources must somehow be apportioned (rationed) among competing users. Empirical analyses of the rationing criteria implicit in treatment decision typically use the logit model to examine the criteria that determine how a specific treatment is rationed. In this paper the analysis is generalised to how scarce resources are rationed in the treatment of a specific disease by selecting more or less aggressive treatment. Our analysis uses the ordered logit model and examines the rationing criteria implicit in the primary treatment choices for non-small-cell lung cancer. Furthermore, our set of explanatory variables includes tumour size, weight loss and smoker status, three variables that appear not to have been used in previous analyses of rationing in the context of NSCLC. We show that the amount of resources apportioned to the primary treatment of a patient's NSCLC declines with a rise in the patient's age, with a deterioration in the patient's functional status and if the patient is an active smoker. Further work is needed to determine why this happens and whether these implicit rationing criteria are directing scarce resources to where they are most beneficial.

Follow-up costs after primary treatment are not included in our analysis. Our focus is on the determinants of resource use at the primary treatment level rather than on the factors that determine resource use by the NSCLC patient from diagnosis to death. Substantial further work is needed to address this much larger issue. Additionally, the robustness of our results need to be explored by estimating ordered logit models for primary treatment selection for NSCLC at other institutions.

### Appendix

Let $T^*$ denote the unobserved willingness to treat a patient more aggressively and let $T_i$ $i = 0,1,2...6$ denote the seven treatment options in this study, ranked from the least aggressive, $T_0$, to most aggressive, $T_6$. Furthermore, let $\mu_0$, $\mu_1 ...$, $\mu_5$ ($\mu_0 < \mu_1 < ... < \mu_6$) denote six threshold values so that if

$$\begin{array}{ll} T^* \leq \mu_0 & T_0 \text{ is chosen} \\ \mu_0 < T^* \leq \mu_1 & T_1 \text{ is chosen} \\ \vdots & \vdots \\ \mu_4 < T^* \leq \mu_5 & T_5 \text{ is chosen} \\ \mu_5 < T^* & T_6 \text{ is chosen} \end{array} \quad (1)$$

The unobserved willingness to treat a patient more aggressively is a continuous variable and is postulated to be a linear function of $K$ observable explanatory variables, i.e.:

$$T^* = \alpha + \sum_{j=1}^{k} \beta_j X_j + \epsilon \quad (2)$$

where $\alpha$ is a constant term, $\beta$ is a vector of coefficients, the $X_j$ are the selected disease indicators and postulated implicit rationing criteria and $\in$ is a disturbance term that is assumed to be logistically distributed.

The ordered logit technique uses a non-linear, maximum likelihood estimation technique, the normalisation $\mu_0 = 0$, and the assumption that $\in$ is logistically distributed to generate estimates of the model parameters, $\mu_1$, $\mu_2, ...,$ $\mu_5$, $\beta_1, ...,$ $\beta_4$, and $a$. These parameter estimates, in conjuncition with specific values for the explanatory variables, $X_j$, can be used to compute the probability that a patient will receive a specified treatment. The formulas for calculating these probabilities are:

$$P_0 = \text{Prob}(T_0 \text{ selected}) = \frac{e^{-\alpha - \sum_{j=1}^{k} \beta_j X_j}}{1 + e^{-\alpha - \sum_{j=1}^{k} \beta_j X_j}}$$

$$P_1 = \text{Prob}(T_1 \text{ selected}) = \frac{e^{\mu_1 - \alpha - \sum_{j=1}^{k} \beta_j X_j}}{\mu_1 - \alpha - \sum^{k} \beta_j X_j} - \frac{e^{-\alpha - \sum_{j=1}^{k} \beta_j X_j}}{-\alpha - \sum^{k} \beta_j X_j}$$

For each set of $X_j$ values there exists a probability distribution across the seven treatment choices.

The expected treatment expenditure for a patient with a given set of $X_j$ values is the weighted sum of the individual costs of the seven treatments, where the weights are the probabilities. An increase in age, holding the other $X$ values constant, will lower the expected expenditure on treatment if it shifts the probability distribution to the left towards less aggressive (less costly) treatment and away from more aggressive (more expensive) treatment. The same is true for a reduction in KPS. Our analysis also tests the hypothesis that, holding all other factors constant, the probability distribution across the seven ordered treatment options sits farther to the right for non- and ex-smokers than for active smokers.

$$P_0 = \text{Prob}(T_0 \text{ selected}) = \frac{e^{-\alpha - \sum_{j=1}^{k} \beta_j X_j}}{1 + e^{-\alpha - \sum_{j=1}^{k} \beta_j X_j}}$$

$$P_1 = \text{Prob}(T_1 \text{ selected}) = \frac{e^{\mu_1 - \alpha - \sum_{j=1}^{k} \beta_j X_j}}{1 + e^{\mu_1 - \alpha - \sum_{j=1}^{k} \beta_j X_j}} - \frac{e^{-\alpha - \sum_{j=1}^{k} \beta_j X_j}}{1 + e^{-\alpha - \sum_{j=1}^{k} \beta_j X_j}}$$

**Acknowledgements**

Supported by the British Columbia Health Research Foundation and the Science Council of British Columbia Health Development Fund Grants HDF 70-89 and 14-90. The authors wish to acknowledge: L Blenkinsop, M Boyle, J Cawsey, S Iwama, R Kitching, E Laukkanen, D Linekin, P McAllister, P Neilson, G Owen, M Saunder, J Schofield, A Stewart, M Thompson, M Wehinger, K Wilson, and I Yong, for their contribution to the design and compilation of the database; R Davidson and D Giles for their helpful suggestions for parts of the statistical analysis; the two anonymous referees for their constructive comments; and the many patients whose cooperation made this study possible. Any errors are our responsibility.

### References

AARON HJ AND SCHWARTZ WB. (1984). *The Painful Prescription: Rationing Hospital Care.* The Brookings Institution: Washington.

BLANCHARD CG, LABRECQUE MS, RUCKDESCHEL JC AND BLANCHARD EB. (1988). Information and decision-making preferences of hospitalized adult cancer patients. *Soc. Sci. Med.*, **27**, 1139–1145.

BUSSCHBACH JJV, HESSING DJ AND DE CHARRO FTH. (1993). The utility of health at different stages in life: a quantitative approach. *Soc. Sci. Med.*, **37**, 153–158.

CANCER CONTROL AGENCY OF BRITISH COLUMBIA. (1988). *Cancer Treatment Policies.* Cancer Control Agency of British Colombia: Vancouver.

CHUTE CG, GREENBERG ER, BARON J, KORSON R, BAKER J AND YATES J. (1985). Presenting conditions of 1539 population-based lung cancer patients by cell type and stage in New Hampshire and Vermont. *Cancer*, **56**, 2107–2111.

COHEN HJ. (1995). Geriatric principles of treatment applied to medical oncology: an overview. *Semin. Oncol.* **22**, (suppl.1), 1–2.

COY P, SCHAAFSMA J, SCHOFIELD JA AND NIELD JA. (1994). Comparative costs of lung cancer management. *Clin. Invest. Med.*, **17**, 577–587.

DETSKY AS, STRICKER SC, MULLEY AG AND THIBOULT GE. (1981). Prognosis, survival, and the expenditure of hospital resources for patients in an intensive-care unit. *N. Engl. J. Med.*, **305**, 667–672.

EVANS RW. (1983*a*). Health care technology and the inevitability of resource allocation and rationing decisions – part 1. *JAMA*, **249**, 2047–2053.

EVANS RW. (1983*b*). Health care technology and the inevitability of resource allocation and rationing decisions – part 2. *JAMA*, **249**, 2208–2219.

FEINSTEIN AR. (1964). Symptomatic patterns, biologic behaviour, and prognosis in cancer of the lung: practical application of boolean algebra and clinical taxonomy. *Ann Intern. Med.*, **61**, 27–43.

GREENBERG ER, CHUTE CG, STUKEL T, BARON JA, FREEMAN DH, YATES J AND KORSON R. (1988). Social and economic factors in the choice of lung cancer treatment. *N. Engl. J. Med.*, **318**, 612–617.

GREENE WH. (1990). *Econometric Analysis*. Macmillan: New York.

GREENE WH. (1992). *LIMDEP Version 6.0*. Econometric Software: Bellport, NY.

GOODWIN JS, HUNT WC, KEY CR AND SAMET JM. (1987). The effect of marital status on stage, treatment, and survival of cancer patients. *JAMA.*, **258**, 3125–3130.

KARNOFSKY DA, ABELMANN WH, CRAVER LF AND BURCHENAL JH. (1948). The use of nitrogen mustards in the palliative treatment of carcinoma. *Cancer*, **1**, 634–656.

KENNEDY P. (1987). *A Guide to Econometrics*, MIT Press: Cambridge.

LIPSCHITZ DA. (1995). Age-related declines in hematopoietic reserve capacity. *Semin. Oncol.*, **22** (suppl.1), 3–5.

LIPWORTH L, ABELIN T AND CONNELLY RR. (1970). Socio-economic factors in the prognosis of cancer patients. *Chron. Dis.*, **23**, 105–116.

MCKELVEY RD AND ZAVOINA W. (1975). A statistical model for the analysis of ordinal level dependent variables. *J. Math. Sociol.*, **4**, 103–120.

MACKILLOP WJ, O'SULLIVAN B AND WARD GK. (1987). Non-small cell lung cancer: how oncologists want to be treated. *Int. J. Rad. Oncol. Biol. Physics.*, **13**, 929–934.

MOR V, MASTERSON-ALLEN S, GOLDBERG RJ, CUMMINGS FJ, GLICKSMAN AS AND FRETWELL MD. (1985). Relationship between age at diagnosis and treatments received by cancer patients. *Amer. Geriatr. Soc.*, **33**, 585–589.

MOUNTAIN CF. (1986). A new international staging system for lung cancer. *Chest*, **89**, 225S–233S.

NAYLOR CD, LEVINTON CM, WHEELER S AND HUNTER L. (1993). Queuing for coronary surgery during severe supply-demand mismatch in a Canadian referral centre: a case study of implicit rationing. *Soc. Sci. Med.*, **37**, 61–67.

SAMET JM, HUNT WC, KEY CR, HUMBLE CG AND GOODWIN JS. (1986). Choice of cancer therapy varies with age of patient. *JAMA*, **255**, 3385–3390.

SCITOVSKY AA. (1988). Medical care in the last twelve months of life: the relation between age, functional status, and medical care expenditures. *Milbank Memorial Fund Quart.*, **66**, 640–660.

SHERMAN S AND GUIDOT CE. (1987). The feasibility of thoracotomy for lung cancer in the elderly. *JAMA*, **258**, 927–930.